# Novel solitons in the (2+1)-dimensional Heisenberg spin chain via generalized conformable derivatives

**DOI:** 10.1038/s41598-025-11975-y

**Published:** 2025-07-20

**Authors:** Badar E Alam, Gesuri Morales-Luna, Guillermo Fernandez-Anaya

**Affiliations:** Department of Physics and Mathematics, Universidad Iberoamericana Ciudad de México, Prolongación Paseo de Reforma 880, Lomas de Santa Fe, Mexico City, 01219 Mexico

**Keywords:** Mathematics and computing, Applied mathematics

## Abstract

This study employs advanced mathematical techniques to investigate exact solutions for the fractional (2+1)-dimensional Heisenberg ferromagnetic spin chain (HFSC) equation. Novel complex transformations—based on the generalized conformable derivative, exponential functions, arctanh, and arctan—are used to reduce the partial differential equation to an ordinary one. Three analytical methods are applied to obtain solutions: the modified Kudryashov method, the improved Bernoulli subequation function method (IBSEFM), and the modified extended direct algebraic method (mEDAM). These methods yield kink-wave, hyperbolic, trigonometric, and periodic wave solutions, which are validated through 2D, 3D, and contour plots for specific parameter choices. The main objective of this study is to derive exact soliton solutions of the Heisenberg spin chain equation using generalized conformable derivatives through multiple analytical methods. A sensitivity analysis is also performed to study how small changes in initial conditions affect the system’s behavior. These findings may contribute to future data storage technologies and magnetic memory developments. The proposed approaches demonstrate efficiency in solving nonlinear fractional equations and have potential applications in shallow water waves, fluid dynamics, quantum mechanics, lattice vibrations in condensed matter, shock wave propagation in plasma, and phase transitions in ferromagnetic materials. The study highlights the effectiveness and reliability of the employed techniques.

## Introduction

Nonlinear models have been applied to explain real-world phenomena, providing valuable insights into complex systems. Among these, fractional nonlinear evolution equations (FNLEEs) are a sophisticated set of differential equations that offer more effective ways to describe such phenomena. These equations are particularly helpful for capturing intricate physical processes, which have attracted considerable interest from researchers. One significant example is the nonlinear Schrödinger equation, which has diverse appeals in areas such as optical fiber systems, quantum mechanics, fluid mechanics, plasma physics, biology, electricity, chemical dispersion, heat flow, shallow water waves, fractal dynamics, and finance^1–3^. Analytical solutions for the 3D fractional WBBM equations have been obtained using conformable derivatives, demonstrating exact traveling-wave solutions that maintain their form during propagation. The solutions exhibit important physical characteristics preserved by the conformable operator framework^4^. The conformable time-fractional Phi-four equation has revealed interactions between different wave types, including lump, periodic, and kink solutions. Detailed dynamical analysis shows these solutions maintain stability during evolution, with the conformable derivative effectively capturing the nonlinear wave interactions^5^. For water wave applications, the conformable space-time fractional (2+1)-dimensional AKNS equation has been analyzed, showing how the derivative operator describes long-wave phenomena. The conformable framework provides accurate modeling of wave propagation characteristics in fluid systems^6^.

Within contemporary magnetic theory, the (2+1)-dimensional HFSCE stands as a fundamental equation for understanding nonlinear magnetic dynamics. Particularly valuable for representing insulating magnetic crystal characteristics and elucidating spin-long ferromagnetic ordered interactions, this equation holds substantial importance in soliton theory investigations. The high-quality soliton solutions it produces have enabled thorough qualitative analyses of numerous phenomena in ferromagnetics, nonlinear optics, and fiber optics. The Heisenberg model’s treatment of ferromagnetic spin chains, with its various magnetic interactions expressed through nonlinear evolution equations, displays particularly orderly behavior when examined through classical and semiclassical continuum approaches^7,8^. A key feature of these equations is the interaction between the nonlinear and dispersive properties of the solitons. As solitons move through a medium, their wave-like structure remains stable. Solutions derived from fractional nonlinear evolution equations have many practical applications, including telecommunications, fiber technology, system modeling, signal and image processing, plasma physics, water purification, medical device sterilization, chemistry, and other fields^9^.

Numerous methods have been proposed and implemented to solve non-linear fractional differential equations (NFDES) and obtain traveling wave solutions. These include the Jacobi elliptic method^10^, the new auxiliary equation method, generalized Kudryashov method, sine-cosine method^11^, modified exp-function method, extended generalized G-expansion method, improved modified extended tanh-function method, Riccati-Bernoulli equation method, new extended direct-algebraic method, sine-Gordon expansion method, inverse scattering transformation method, Lie symmetry analysis method, Bäcklund transformation method, unified algebraic method, homogeneous balance method, the exp-function method, and the modified simple equation method. These approaches have been extensively studied in the literature^12–19^.

Fractional derivatives have shown their importance in many scientific and engineering disciplines, such as physics, signal processing, mechanics, control systems, biomedical engineering, economics, electromagnetism, finance, and fluid mechanics. For example, fractional calculus models the stress and relaxation behavior of viscoelastic food ingredients. This highlights the fractional derivatives’ practical and versatile value in modeling complex systems, enabling more accurate representations and a deeper understanding of various phenomena. Fractional calculus has gained remarkable attention due to its ability to model real-world problems involving memory and hereditary properties. It provides a robust framework for studying nonlinear systems in various fields, such as shallow water waves, fluid dynamics, optical fibers, and biological processes^20^. Among the many nonlinear equations, the (2+1)-dimensional (HFSC) equation is particularly notable for describing magnetic interactions in semiclassical and classical limits^21^. As a key theoretical model in condensed matter physics, the Heisenberg ferromagnet describes how certain materials maintain spontaneous magnetic ordering at reduced temperatures^22^. The HFSC equation governs nonlinear spin wave propagation in ferromagnets, exhibiting solitons and chaos while connecting classical and quantum spin dynamics. Its study enhances spintronics, quantum materials, and nonlinear field theory, demonstrating profound theoretical and applied significance^23^.

The fractional (2+1)-dimensional HFSC equation extends the classical model by incorporating fractional derivatives. This generalization allows a more accurate representation of physical phenomena, such as wave propagation in complex media^24^. However, solving such equations analytically poses significant challenges due to their complexity.

In recent years, several mathematical techniques have been developed to tackle nonlinear fractional equations. These include the inverse scattering transform^25^, Darboux transformation^26^, and the homotopy perturbation method^27^. In this work, we focus on modified Kudryashov method^29^, IBSEFM^30,31^ and (mEDAM)^32^. These methods are applied to the fractional HFSC equation to derive various wave solutions and kink waves, including dark solitons, periodic waves, and singular solutions. Mahdi et al.^33^ analytically solved the (1+1)-dimensional chiral nonlinear Schrödinger equation using an extended direct algebraic method. Their work derived novel soliton solutions, including bright, dark, and periodic waves, validated through 3D visualizations. The study provides insights into wave propagation in nuclear and optical systems, demonstrating the method’s effectiveness for fractional Partial Differential Equations (PDEs).

Recent advances in nonlinear dynamics have highlighted the critical importance of sensitivity analysis for understanding solution stability in fractional-order systems. Nadeem et al.^34^ demonstrated that Beta-derivative formulations exhibit remarkable robustness to parameter variations in Schrödinger models, with soliton solutions maintaining structural integrity despite significant perturbations. Their work established that RK4-based sensitivity analysis provides crucial insights into optical soliton stability in fiber optic applications. Complementing this, Nasreen et al.^35^ implemented phase-space sensitivity techniques for Boussinesq equations, revealing minimal solution divergence under initial condition variations. Their graphical analysis of phase portraits established new benchmarks for assessing dynamical stability in water wave models.

Building on these foundations, Amin et al.^36^ systematically compared solution profiles across fractional orders $\sigma$, demonstrating that amplitude variations follow predictable scaling laws in higher-order Schrödinger systems. Their bifurcation analysis further confirmed that sensitivity peaks near critical parameter thresholds. Yang et al.^37^ expanded this paradigm to chaotic Zoomeron models, developing perturbation-response frameworks that quantify solution resilience. Collectively, these studies establish sensitivity analysis as indispensable for validating nonlinear wave phenomena across physics and engineering domains, particularly for systems exhibiting fractional dynamics and memory effects.

The novelty of this study lies in the formulation of wave transformations using generalized conformable derivatives, along with the introduction of a new transformation function designed to simplify the analytical structure of the Heisenberg Spin Chain equation. We employ three distinct auxiliary functions and apply three advanced analytical methods—namely, the Kudryashov method, the improved Bernoulli sub-equation function method (IBSEFM), and the modified extended direct algebraic method (mEDAM)—to derive a wide variety of exact soliton solutions, including kink, periodic, singular, and bright-dark solitons. Symbolic derivations and solution construction were performed using Maple, while all graphical visualizations were rendered using Mathematica. This integrated framework not only broadens the class of solvable nonlinear models but also provides deeper physical insight into the dynamics of integrable spin systems, surpassing the scope of earlier methods. The relevance of generalized conformable derivatives is that they turn a Partial Differential Equation (PDE) into a family of PDEs. A specific generalized conformable derivative is determined by the function that multiplies the integer-order derivative, and this function depends on the independent variable and the order of the derivative. Therefore, there are different PDEs for different generalized conformable derivatives, starting from a PDE of integer order, and different solutions for each of these. Consequently, these derivatives allow for the discovery of solutions to new PDEs, generated from integer-order PDEs, which can be interpreted as deformations of integer-order PDEs, by modifying the coefficients of the integer-order PDEs via the generalized conformable derivatives. The structure of this document is as follows: “Basic one-dimensional GCD results” introduces the definition and some remarks. “Methods” presents the approach and a description of the methodologies. “Application of the methods” presents the mathematical model and application of these techniques to the fractional HFSC model, as well as the solutions and their graphical representations. “Sensitivity analysis” shows a sensitivity analysis and discussion and graphical behavior in “Results, graphical representations and discussion”; “Conclusion” summarizes the results and recommendations for further research.

## Basic one-dimensional GCD results

This section covers the General Conformable Derivatives (GCD) definition and characteristics covered in^38^.

**Definition of GCFD ** The GCFD is based on Linear Extended Gâteaux Derivative (LEGD). Let *Y* and *X* be locally convex vector spaces topologically, and let $g: X \rightarrow Y$. The LEGD of *g* at $\upsilon \in X$ is defined as follows:

1 $$\begin{aligned} d g(\upsilon ; \Phi ) = \lim _{\epsilon \rightarrow 0} \frac{g(\upsilon + \epsilon \Phi (\upsilon , \alpha )) - g(\upsilon )}{\epsilon }, \end{aligned}$$

where $\Phi (\upsilon , \alpha )$ is a function satisfying certain conditions^38^.

The GCFD for a function $g: \mathbb {R}^+ \rightarrow \mathbb {R}$ is defined as:

2 $$\begin{aligned} D^\alpha _\Phi g(\upsilon ) = \lim _{\epsilon \rightarrow 0} \frac{g(\upsilon + \epsilon \Phi (\upsilon , \alpha )) - g(\upsilon )}{\epsilon }, \end{aligned}$$

where $\Phi (\upsilon , \alpha )$ is a conformable function.

### Definition 1

(LEGD). Consider the following: $\Phi (\upsilon , \alpha ) : X \times \mathbb {R} \rightarrow X$, and $f : X \rightarrow Y$, where $\alpha \in \mathbb {R}$, $U \subset X$ is open, and *Y* and *X* are topological locally convex vector spaces. The following is definition of the LEGD $dg(\upsilon ;\Phi )$ of *g* at $\upsilon \in U$:

3 $$\begin{aligned} dg(\upsilon ;\Phi ) = \lim _{\epsilon \rightarrow 0} \frac{g(\upsilon + \epsilon \Phi (\upsilon , \alpha )) - g(\upsilon )}{\epsilon }, \end{aligned}$$

if the limit exists.

### Remark 2

Let *Y*, *X* be Euclidean spaces $\mathbb {R}^m$ and $\mathbb {R}^n$, respectively, and let $g: X \rightarrow Y$ and $\Phi (\upsilon , \alpha ): X \times \mathbb {R} \rightarrow X$. This is an important property of the LEGD. For every *i*, *j*, if $\frac{\partial g_j}{\partial \upsilon _i}$ exists, then:

4 $$\begin{aligned} dg(\upsilon ;\Phi ) = \big (\langle \nabla g_1, \Phi (\upsilon , \alpha ) \rangle , \langle \nabla g_2, \Phi (\upsilon , \alpha ) \rangle , \dots , \langle \nabla g_m, \Phi (\upsilon , \alpha ) \rangle \big ). \end{aligned}$$

### Definition 3

(Fractional Conformable Function). If a function $\Phi (\upsilon , \alpha )$ meets the following criteria, it is referred to as a fractional conformable function:

1. $$\Phi (\upsilon , \alpha ) \ne 0, \quad u \in \mathbb {R}^+$$;
2. $$\Phi (\upsilon , 1) = 1, \quad u \in \mathbb {R}^+$$;
3. $$\Phi (\cdot , \beta )\ne \Phi (\cdot , \alpha ), \quad \text {for } \beta , \alpha \in (0, 1], \alpha \ne \beta .$$
4. $$\Phi (\upsilon , \alpha )$$ is differentiable.
5. $$\Phi (\upsilon , \alpha )$$ is positive.

### Definition 4

(GCFD). Let $\alpha \in (0, 1]$ and $\Phi (\upsilon , \alpha )$ be fractional conformable functions. The following is the definition of the GCFD of $g: \mathbb {R}^+ \rightarrow \mathbb {R}$ at $\upsilon$:

5 $$\begin{aligned} D^\alpha _\Phi g(\upsilon ) = \lim _{\epsilon \rightarrow 0} \frac{g(\upsilon + \epsilon \Phi (\upsilon , \alpha )) - g(\upsilon )}{\epsilon }. \end{aligned}$$

### Remark 5

If $\Phi (\upsilon , \alpha ) = 1$, then $D^\alpha _\Phi f(\upsilon )$ reduces to the classical first-order derivative and has no dependence on $\alpha$.

### Remark 6

If $\Phi (\upsilon , \alpha ) = \upsilon ^{1-\alpha }$, then $D^\alpha _\Phi f(\upsilon )$ coincides with the conformable derivative proposed by Khalil et al.^39^.

### Definition 7

(Left GCFD). For *g* defined on $[a, \infty )$, the left GCFD is defined as:

6 $$\begin{aligned} D^\alpha _\Phi g(\upsilon ) = \lim _{\epsilon \rightarrow 0} \frac{g(\upsilon + \epsilon \Phi (\upsilon -a, \alpha )) - g(\upsilon )}{\epsilon }. \end{aligned}$$

If the limit exists. The left GCFD generalizes the conformable derivative on the left defined by^40^.

### Theorem 8

*Assume that* *g*, *f* *are* $\alpha$-*differentiable at* $\upsilon > 0$ *and that* $\alpha \in (0, 1]$. *Then*:

1. *Linearity*: $$D^\alpha _\Phi (bg+af) = bD^\alpha _\Phi (g)+aD^\alpha _\Phi (f), \quad \forall ~ b,a \in \mathbb {R}$$.
2. *Constant rule*: $D^\alpha _\Phi (c) = 0$, *c* *is any constant*.
3. *Product rule*: $$D^\alpha _\Phi (gf) = gD^\alpha _\Phi (f) + fD^\alpha _\Phi (g)$$.
4. *Quotient rule*: $$D^\alpha _\Phi \left( \frac{g}{f}\right) = \frac{fD^\alpha _\Phi (g) - gD^\alpha _\Phi (f)}{f^2}$$.
5. *If* *g* *is differentiable*, $D^\alpha _\Phi (g) = \frac{dg}{du} \Phi (\upsilon , \alpha )$.
6. *Chain rule*: $$D^\alpha _\Phi (g \circ f(\upsilon )) = g'(f(\upsilon )) D^\alpha _\Phi (f(\upsilon ))$$.

### Definition 9

(GCFD of Arbitrary Order). The arbitrary order GCFD is defined as follows for $\alpha \in (n, n+1]$, $n \in \mathbb {N}$, with *g* being *n*-differentiable at $\upsilon > 0$:

7 $$\begin{aligned} D^\alpha _\Phi g(\upsilon ) = D^{\alpha -n}_\Phi \big (D^n g(\upsilon )\big )=\lim _{\epsilon \rightarrow 0} \frac{g^n(\upsilon + \epsilon \Phi (\upsilon , \alpha -n)) - g^{(n)}(\upsilon )}{\epsilon }. \end{aligned}$$

If the limit exists^38^.

## Methods

Consider the non-linear time fractional conformable differential equation as follows^29^:

8 $$\begin{aligned} {\Phi \left( \frac{\partial ^\beta u}{\partial t^\beta }, u_x, u_{xx}, \frac{\partial ^\beta u_x}{\partial t^\beta }, u_{yy}, \dots \right) = 0}, \end{aligned}$$

where the conformable-order derivative is represented by $\beta$ and $u$ is a function of $x$ and $t$. The novel wave transformation that we developed and used in this instance is the following:

9 $$\begin{aligned} {u(x, y, t) = e^{(i\theta )}~ U(\eta ),~~~\theta = x+y-\omega \int \frac{dt}{\Phi (t,\beta )},~\theta = x+y+\nu \int \frac{dt}{\Phi (t,\beta )}}. \end{aligned}$$

In particular, we use 1. $\Phi (t,\beta )=\exp ((1-\beta )t)$, 2.$\Phi (t,\beta )=(1-\beta )t^2+1,$ 3.$\Phi (t,\beta )=\sin ^2(1-\beta )t+1.$ With this new wave transformation applied to Eq. (8), a couple of nonlinear ordinary differential equations result:

10 $$\begin{aligned} {\Phi \left( U, U', U'', U''', \dots \right) = 0}. \end{aligned}$$

### Modified Kudryashov procedure

For the modified Kudryashov method^28,29^, we select $U(\eta )$ as:

11 $$\begin{aligned} {U(\eta ) = \sum _{i=0}^{n} a_i Z^i(\eta )}, \end{aligned}$$

where $$Z(\eta )$$ satisfies:

12 $$\begin{aligned} {\frac{d Z}{d \eta } = \left( Z^2(\eta ) - Z(\eta ) \right) \ln \omega }. \end{aligned}$$

The solution to Eq. (12) is:

13 $$\begin{aligned} {Z(\eta ) = \frac{1}{1 + \lambda \omega ^\eta }}. \end{aligned}$$

Replacement of Eq. (11) into Eq. (10) and solving the resulting algebraic system, the values of $a_i$, $v$, are determined, allowing us to construct the exact solutions.

#### Remark

The solutions derived here are specific cases of generalized Kudryashov method solutions. The chosen auxiliary equations make our methods unique compared to others in the literature.

### mEDAM methodology

This section explains the methodology of mEDAM, which is used to solve fractional partial differential equations (FPDEs). Consider an FPDE of the general form:

14 $$\begin{aligned} {\Phi \left( \frac{\partial ^\beta u}{\partial t^\beta }, u_x, u_{xx}, \frac{\partial ^\beta u_x}{\partial t^\beta }, u_{yy}, \dots \right) = 0}, \quad 0 < \beta \le 1, \end{aligned}$$

The function $\Phi$ depends on $y_1, y_2, \dots ,y_r,x_1, x_2, \dots , x_r$, and *t*. The following steps outline the solution procedure:

1. Transformation of variables: Transform the dependent variable *u*(*t*, *x*, *y*) into $U(\eta )$ using a traveling wave transformation, where $\eta$ combines the independent variables. This transformation reduces the FPDE into a non-linear ordinary differential equation (ODE):
  15 $$\begin{aligned} \Phi \big (U, U', U'', \dots \big ) = 0, \end{aligned}$$
  The derivative of *U* with respect to $\eta$ is shown by $U'$. Integration of this ODE may introduce constants of integration.
2. Solution assumption: Assume that the solution to the ODE can be expressed in the following series form:
  16 $$\begin{aligned} W(\eta ) = \sum _{l=-m}^{m} d_l \big (Z(\eta )\big )^l, \end{aligned}$$
  where $Z(\eta )$ satisfies the auxiliary equation and $d_l$ are constants that need to be found:
  17 $$\begin{aligned} \frac{dZ(\eta )}{d\eta )} = \big (a + b Z(\eta ) + c Z^2(\eta )\big )\ln (\varkappa ) , \end{aligned}$$
  with constants *a*, *b*, *c*, and $\varkappa \ne 1,0$.
3. Determination of *m*: To find the positive integer *m*, the highest derivative of the ODEs and the highest-order nonlinear term must be balanced. This establishes the degree of the solution series.
4. Substitution into the ODE: Collect terms with the same powers as $Z(\eta )$ and replace the assumed solution in the ODE. To create a system of equations for $d_l$ and other parameters, set the coefficients of like powers to zero.
5. Solution of algebraic equations: Use symbolic computing tools such as Mathematica or Maple to solve the resulting system of algebraic equations.
6. Final solution: Substitute the solved values of $d_l$ and the solution of $Z(\eta )$ back into the series expression to obtain the final analytical solution to the original FPDE.

## Family of solitary wave solutions

This section presents a methodology that uses mEDAM to derive families of solitary waves for the fractional HFSC system. We find different cases by placing different conditions on different parameters. Here are comprehensive answers arranged by family:

### Family 01

For $S< 0$ and $c \ne 0$, the solutions are given as follows:

$$\begin{aligned} Z_{01}(\eta )&= -\frac{b}{2c} + \frac{\sqrt{-S} \tan _\varkappa \left( \frac{1}{2}\sqrt{-S}\eta \right) }{2c},\\ Z_{02}(\eta )&= -\frac{b}{2c} - \frac{\sqrt{-S} \cot _\varkappa \left( \frac{1}{2}\sqrt{-S}\eta \right) }{2c},\\ Z_{03}(\eta )&= -\frac{b}{2c} + \frac{\sqrt{-S}\left( \tan _\varkappa \left( \sqrt{-S}\eta \right) \pm \sqrt{p_1q_2} \sec _\varkappa \left( \sqrt{-S}\eta \right) \right) }{2c}, \\ Z_{04}(\eta )&= -\frac{b}{2c} - \frac{\sqrt{-S}\left( \cot _\varkappa \left( \sqrt{-S}\eta \right) \pm \sqrt{p_1q_2} \csc _\varkappa \left( \sqrt{-S}\eta \right) \right) }{2c}, \\ Z_{05}(\eta )&= -\frac{b}{2c} + \frac{\sqrt{-S}\left( \tan _\varkappa \left( \frac{1}{4}\sqrt{-S}\eta \right) - \cot _\varkappa \left( \frac{1}{4}\sqrt{-S}\eta \right) \right) }{4c}. \end{aligned}$$

### Family 02

For $c \ne 0$ and $S > 0$, solutions are given as:

$$\begin{aligned} Z_{06}(\eta )&= -\frac{b}{2c} - \frac{\sqrt{S} \tanh _\varkappa \left( \frac{1}{2}\sqrt{S}\eta \right) }{2c}, \\ Z_{07}(\eta )&= -\frac{b}{2c} - \frac{\sqrt{S} \coth _\varkappa \left( \frac{1}{2}\sqrt{S}\eta \right) }{2c},\\ Z_{08}(\eta )&= -\frac{b}{2c} - \frac{\sqrt{S}\left( \tanh _\varkappa \left( \sqrt{S}\eta \right) \pm \sqrt{p_1q_2} \operatorname {sech}_\varkappa \left( \sqrt{S}\eta \right) \right) }{2c}, \\ Z_{09}(\eta )&= -\frac{b}{2c} - \frac{\sqrt{S}\left( \coth _\varkappa \left( \sqrt{S}\eta \right) \pm \sqrt{p_1q_2} \operatorname {csch}_\varkappa \left( \sqrt{S}\eta \right) \right) }{2c}, \\ Z_{10}(\eta )&= -\frac{b}{2c} - \frac{\sqrt{S}\left( \tanh _\varkappa \left( \frac{1}{4}\sqrt{S}\eta \right) - \coth _\varkappa \left( \frac{1}{4}\sqrt{S}\eta \right) \right) }{4c}. \end{aligned}$$

### Family 03

For $b = 0$ and $ac > 0$, the solutions are:

$$\begin{aligned} Z_{11}(\eta )&= \sqrt{\frac{a}{c}} \tan _\varkappa \left( \sqrt{ac}\eta \right) ,\\ Z_{12}(\eta )&= -\sqrt{\frac{a}{c}} \cot _ \varkappa \left( \sqrt{ac}\eta \right) , \\ Z_{13}(\eta )&= \sqrt{\frac{a}{c}} \left( \tan _\varkappa \left( 2\sqrt{ac}\eta \right) \pm \sqrt{p_1q_2} \sec _\varkappa \left( 2\sqrt{ac}\eta \right) \right) , \\ Z_{14}(\eta )&= -\sqrt{\frac{a}{c}} \left( \cot _\varkappa \left( 2\sqrt{ac}\eta \right) \pm \sqrt{p_1q_2} \csc _\varkappa \left( 2\sqrt{ac}\eta \right) \right) , \\ Z_{15}(\eta )&= \frac{1}{2}\sqrt{\frac{a}{c}}\left( \tan _\varkappa \left( \frac{1}{2}\sqrt{ac}\eta \right) - \cot _ \varkappa \left( \frac{1}{2}\sqrt{ac}\eta \right) \right) . \end{aligned}$$

### Family 04

For $b = 0$ and $ac < 0$, the solutions are:

$$\begin{aligned} Z_{16}(\eta )&= -\sqrt{-\frac{a}{c}} \tanh _\varkappa \left( \sqrt{-ac}\eta \right) , \\ Z_{17}(\eta )&= -\sqrt{-\frac{a}{c}} \coth _\varkappa \left( \sqrt{-ac}\eta \right) , \\ Z_{18}(\eta )&= -\sqrt{-\frac{a}{c}} \left( \tanh _\varkappa \left( 2\sqrt{-ac}\eta \right) \pm i\sqrt{p_1q_2} \operatorname {sech}_\varkappa \left( 2\sqrt{-ac}\eta \right) \right) , \\ Z_{19}(\eta )&= -\sqrt{-\frac{a}{c}} \left( \coth _\varkappa \left( 2\sqrt{-ac}\eta \right) \pm \sqrt{p_1q_2} \operatorname {csch}_\varkappa \left( 2\sqrt{-ac}\eta \right) \right) , \\ Z_{20}(\eta )&= -\frac{1}{2}\sqrt{-\frac{a}{c}}\left( \tanh _\varkappa \left( \frac{1}{2}\sqrt{-ac}\eta \right) + \coth _\varkappa \left( \frac{1}{2}\sqrt{-ac}\eta \right) \right) . \end{aligned}$$

### Family 05

For $b = 0$ and $c = a$, the solutions are:

$$\begin{aligned} Z_{21}(\eta )&= \tan _ \varkappa (a\eta ), \\ Z_{22}(\eta )&= -\cot _\varkappa (a\eta ),\\ Z_{23}(\eta )&= \tan _\varkappa (2a\eta ) \pm \sqrt{p_1q_2} \sec _\varkappa (2a\eta ), \\ Z_{24}(\eta )&= -\cot _\varkappa (2a\eta ) \pm \sqrt{p_1q_2} \csc _\varkappa (2a\eta ),\\ Z_{25}(\eta )&= \frac{1}{2}\left( \tan _\varkappa \left( \frac{1}{2}a\eta \right) - \cot _\varkappa \left( \frac{1}{2}a\eta \right) \right) . \end{aligned}$$

### Family 06

For $b = 0$ and $c = -a$, the solutions are:

$$\begin{aligned} Z_{26}(\eta )&= -\tanh _\varkappa (a\eta ), \\ Z_{27}(\eta )&= -\coth _ \varkappa (a\eta ), \\ Z_{28}(\eta )&= -\tanh _\varkappa (2a\eta ) \pm i\sqrt{p_1q_2} \operatorname {sech} _\varkappa (2a\eta ), \\ Z_{29}(\eta )&= -\coth _ \varkappa (2a\eta ) \pm \sqrt{p_1q_2} \operatorname {csch}_\varkappa (2a\eta ), \\ Z_{30}(\eta )&= -\frac{1}{2}\left( \tanh _\varkappa \left( \frac{1}{2}a\eta \right) + \coth _\varkappa \left( \frac{1}{2}a\eta \right) \right) . \end{aligned}$$

### Family 07

For $S = 0$, the solutions are:

$$\begin{aligned} Z_{31}(\eta )&= -\frac{2a}{b^2 \eta \ln (\varkappa ) + 4}. \end{aligned}$$

### Family 08

For $c = 0$, $b = \lambda$ and $a = n\lambda$ ($n \ne 0$), the solutions are:

$$\begin{aligned} Z_{32}(\eta )&= \varkappa ^{\lambda \eta } - n. \end{aligned}$$

### Family 09

For $c = b = 0$, the solutions are:

$$\begin{aligned} Z_{33}(\eta )&= a\eta \ln (\varkappa ). \end{aligned}$$

### Family 10

For $b = a = 0$, the solutions are:

$$\begin{aligned} Z_{34}(\eta )&= -\frac{1}{c\eta \ln (\varkappa )}. \end{aligned}$$

### Family 11

For $c \ne 0$, $a = 0$ and $b \ne 0$, the solutions are:

$$\begin{aligned} Z_{35}(\eta )&= -\frac{b}{c(\cosh _\varkappa (b\eta ) - \sinh _\varkappa (b\eta ) + p_1)},\\ Z_{36}(\eta )&= -\frac{b(\cosh _\varkappa (b\eta ) + \sinh _ \varkappa (b\eta ))}{c(\cosh _\varkappa (b\eta ) + \sinh _\varkappa (b\eta ) + q_1)}. \end{aligned}$$

### Family 12

For $c = n\lambda$ ($n \ne 0$), $b = \lambda$, and $a = 0$, the solutions are:

$$\begin{aligned} Z_{37}(\eta )&= \frac{p_1 \varkappa ^{\lambda \eta }}{p_1 - n q_1 \varkappa ^{\lambda \eta }}. \end{aligned}$$

### Generalized hyperbolic and trigonometric functions

The generalized hyperbolic function and trigonometric functions used in the solutions are:

$$\begin{aligned} \cos _ \varkappa (\eta )&= \frac{p_1 \varkappa ^{i \eta } + q_1 \varkappa ^{-i \eta }}{2},&\sin _\varkappa (\eta )&= \frac{p_1 \varkappa ^{i \eta } - q_1 \varkappa ^{-i \eta }}{2i}, \\ \cosh _\varkappa (\eta )&= \frac{p_1 \varkappa ^{\eta } + q_1 \varkappa ^{-\eta }}{2},&\sinh _\varkappa (\eta )&= \frac{p_1 \varkappa ^{\eta } - q_1 \varkappa ^{-\eta }}{2}. \end{aligned}$$

### Family-specific behavior

Each family of solutions represents different wave profiles, such as kink waves, lump waves, and periodic solitons. These profiles are critical in understanding the dynamics of fractional systems.

### An explanation of the IBSEFM approach

The basic characteristics of IBSEFM are listed in this section^30,31^. The IBSEFM consists of the following five primary steps:

Step 1: Using space *x* and time *t* as variables, let us examine the following equation with the conformable derivative for a function:

18 $$\begin{aligned} { \Phi \left( \frac{\partial ^\beta u}{\partial t^\beta }, u_x, u_{xx}, \frac{\partial ^\beta u_x}{\partial t^\beta }, u_{yy}, \dots \right) = 0}, \end{aligned}$$

where $\Phi$ includes partial derivatives and *u*(*x*, *y*, *t*). The objective is to use an appropriate wave transformation to convert Eq. (18) to a nonlinear ODE as

19 $$\begin{aligned} { u(x, y, t) = e^{(i\theta )}~ U(\eta ),~~~\theta = x+y-\omega \int \frac{dt}{\Phi (t,\beta )},~\eta = x+y+\nu \int \frac{dt}{\Phi (t,\beta )}}. \end{aligned}$$

Here, the constants $\omega$ and $\nu$ are arbitrary. When Eq. (19) is used, Eq. (18) becomes the ODE in the following form:

20 $$\begin{aligned} \Phi (U, U', U'', ...) = 0, \end{aligned}$$

where $\Phi$ is the function of *U*, $U'$, $U''$, ... and its derivatives with respect to $\eta$. We obtain integration constants by integrating Eq. (20) term by term; these can be determined later.

Step 2: We speculate that the following is one possible presentation of the solution to Eq. (20):

21 $$\begin{aligned} U(\eta ) = \frac{\sum _{i=0}^n a_i Z^i(\eta )}{\sum _{j=0}^m b_j Z^j(\eta )} = \frac{a_0 + a_1 Z(\eta ) + a_2 Z^2(\eta ) + ... + a_n Z^n(\eta )}{b_0 + b_1 Z(\eta ) + b_2 Z^2(\eta ) + ... + b_m Z^m(\eta )}, \end{aligned}$$

where $b_0, b_1,..., b_m$ and $a_0, a_1,..., a_n$ are coefficients to be determined late, $n \ne 0$, $m \ne 0$ are chosen arbitrarily according to the balancing principle, and $Q\eta )$ satisfies the Bernoulli differential equation:

22 $$\begin{aligned} Z'(\eta ) = \sigma Z(\eta ) + d Z^S(\eta ), \quad \sigma \ne 0, ~d \ne 0, ~ S \in \mathbb {R} \setminus \{0, 1, 2\}. \end{aligned}$$

Step 3: The balancing principle, which incorporates both the highest-order derivative term and the nonlinear term of Eq. (20), is used to determine the positive integers *n*, *m* and *S*. We obtain a polynomial equation $\Omega (Z)$ in *Z* substituting Eq. (21) and Eq. (22) into Eq. (20) as follows:

23 $$\begin{aligned} \Omega (Z(\eta )) = \alpha Z(\eta )^s + ... + \alpha _1 Z(\eta ) + \alpha _0 = 0, \end{aligned}$$

where $\alpha _i$ are the coefficients to be determined later.

Step 4: The coefficients of $\Omega (Q(\eta ))$ provide us with an algebraic system:

24 $$\begin{aligned} \alpha _i = 0, \quad i = 0, ..., s. \end{aligned}$$

Step 5: Solving Eq. (22), with regard to *d* and $\sigma$, we obtain the two examples shown below:

25 $$\begin{aligned} Z(\eta )&= \Bigg [-d e^{\sigma (\epsilon - 1)\eta } + \frac{\epsilon \sigma }{\sigma e^{\sigma (\epsilon - 1)\eta }}\Bigg ]^{\frac{1}{1-\epsilon }}, \quad d \ne \sigma , \end{aligned}$$

26 $$\begin{aligned} Z(\eta )&= \Bigg [\frac{(\epsilon + 1) \tanh \big (\frac{\sigma (1 - \epsilon )\eta }{2}\big )+(\epsilon - 1) }{1 - \tanh \big (\frac{\sigma (1 - \epsilon )\eta }{2}\big )}\Bigg ], \quad d = \sigma , ~~\epsilon \in \mathbb {R}. \end{aligned}$$

Maple is used to generate and categorize precise solutions of Eq. (18) using the entire discrimination system for polynomials of $Z(\eta )$. Plotting 2D and 3D graphs showing precise answers for appropriate parameter values might improve the results.

## Mathematical analysis of the model

Consider the non-linear fractional (2+1) dimensional HFSC equation^41^ with the generalized fractional conformable derivative as follows:

27 $$\begin{aligned} { iD^\beta _t u + A u_{xx}+ B u_{yy} + C u_{xy}- F |u|^2 u = 0, \quad 0< \beta < 1}, \end{aligned}$$

where A, B, C, F are constant and $u=u(x,y,t)$ is a complex function of HFSC, *y* and *x* represent spatial coordinates and *t* represents time coordinates, respectively. $D^\beta _t u$ is GCFD of *u* of order $\beta$.

We now use the transformation.

28 $$\begin{aligned} { u(x, t) = e^{i\theta } U(\eta ), \quad \theta = x+y-\omega {\frac{\,{\textrm{e}^{ \left( 1-\beta \right) t}}}{1-\beta }}, \quad \eta = x+y+\nu \ {\frac{\,{\textrm{e}^{ \left( 1-\beta \right) t}}}{1-\beta }}} , \end{aligned}$$

or

29 $$\begin{aligned} \theta = x+y-\omega \frac{ \textrm{arctanh}\! \left( \sqrt{1-\beta }\, t\right) }{\sqrt{1-\beta }},~~ \eta = x+y+\nu \frac{ \textrm{arctanh}\! \left( \sqrt{1-\beta }\, t\right) }{\sqrt{1-\beta }}, \end{aligned}$$

or

30 $$\begin{aligned} \theta = x+y-\omega \frac{ \textrm{arctan}\! \left( \sqrt{2}\tan (1-\beta )\, t\right) }{\sqrt{2}(1-\beta )}, ~~\eta =x+y+\nu \frac{ \textrm{arctan}\! \left( \sqrt{2}\tan (1-\beta )\, t\right) }{\sqrt{2}(1-\beta )}. \end{aligned}$$

Here, $\omega$, $\nu$, $\theta$, are the velocity, frequency, and phase components, respectively. By substituting Eq. (28), (29) or (30) to Eq. (27), we obtain a system of equations in real and imaginary form.

From the imaginary part, we have $\nu =2A+2B+2C$, and from the real part, we get

31 $$\begin{aligned} { (C+B+A) U'' - (C+B+A+\omega ) U -FU^3 = 0}. \end{aligned}$$

## Application of the methods

In this section, we apply these three methods to find exact solutions of the HFSC equation.

### Modified Kudryashov method

In this subsection, we determine the HFSC’s solitary wave solution using a modified Kudryashov approach. By balancing terms $U^3$ and $U''$ in Eq. (31), we get $n=1$. Using Eq. (11), the assume solution of Eq. (31) becomes:

32 $$\begin{aligned} U(\eta ) = a_0 + a_1 Z. \end{aligned}$$

Upon replacing Eq. (32) with Eq. (12) in Eq. (31), we discover a system of polynomials. By setting the coefficients to zero, we obtain the following system of equations:

33 $$\begin{aligned}&2 A \ln \! \left( \varkappa \right) ^{2} a_{1}+2 B \ln \! \left( \varkappa \right) ^{2} a_{1}+2 C \ln \! \left( \varkappa \right) ^{2} a_{1}-F a_{1}^{3}=0,\nonumber \\&\quad -3 A \ln \! \left( \varkappa \right) ^{2} a_{1}-3 B \ln \! \left( \varkappa \right) ^{2} a_{1}-3 C \ln \! \left( \varkappa \right) ^{2} a_{1}-3 F a_{0} a_{1}^{2}=0,,\nonumber \\&\quad A \ln \! \left( \varkappa \right) ^{2} a_{1}+B \ln \! \left( \varkappa \right) ^{2} a_{1}+C \ln \! \left( \varkappa \right) ^{2} a_{1}-3 F a_{0}^{2} a_{1}-A a_{1}-B a_{1}-C a_{1}-\omega a_{1}=0,\nonumber \\&\quad -F a_{0}^{3}-A a_{0}-B a_{0}-C a_{0}-\omega a_{0}=0. \end{aligned}$$

After solving this system of equations for the unknown $a_{o},a_{1},\omega$, we get

34 $$\begin{aligned} \omega&= -\frac{A \ln \! \left( \varkappa \right) ^{2}}{2}-\frac{B \ln \! \left( \varkappa \right) ^{2}}{2}-\frac{C \ln \! \left( \varkappa \right) ^{2}}{2}-A-B-C ,\nonumber \\ d_{0}&=\sqrt{-\frac{-A-B-C}{2 F}}\, \ln \! \left( \varkappa \right) ,a_{1} = -\frac{\ln \! \left( \varkappa \right) \left( C+B+A\right) }{F \sqrt{-\frac{-A-B-C}{2 F}}},\nonumber \\ a_{0}&=\sqrt{-\frac{-A-B-C}{2 F}}\, \ln \! \left( \varkappa \right) . \end{aligned}$$

The solution of Eq. (27) is obtained by adding these coefficients to Eq. (13) in Eq. (32).

35 $$\begin{aligned} u_1(x,y,t)= \left( \frac{\sqrt{-\frac{-2 A-2 B-2 C}{F}}\, \ln \! \left( \varkappa \right) }{2}-\frac{2 \ln \! \left( \varkappa \right) \left( C+B+A\right) }{\left( 1+\chi \,\omega ^{x+y+\nu \frac{ {\mathrm e}^{\left( 1-\beta \right) t}}{1-\beta }}\right) F \sqrt{-\frac{-2 A-2 B-2 C}{F}}}\right) {\mathrm e}^{\textrm{I} \left( x+y-\omega \frac{ {\mathrm e}^{\left( 1-\beta \right) t}}{1-\beta }\right) }. \end{aligned}$$

The solution of Eq. (27) is obtained by adding these coefficients to Eq. (13) in Eq. (32). We use transformation (29).

36 $$\begin{aligned} u_2(x,y,t)= \left( \frac{\sqrt{-\frac{-2 A-2 B-2 C}{F}}\, \ln \! \left( \varkappa \right) }{2}-\frac{2 \ln \! \left( \varkappa \right) \left( C+B+A\right) }{\left( 1+\chi \,\omega ^{x+y+\nu \frac{ \textrm{arctanh}\! \left( \sqrt{1-\beta }\, t\right) }{1-\beta }}\right) F \sqrt{-\frac{-2 A-2 B-2 C}{F}}}\right) {\mathrm e}^{\textrm{I} \left( x+y-\omega \frac{ \textrm{arctanh}\! \left( \sqrt{1-\beta }\, t\right) }{1-\beta }\right) }. \end{aligned}$$

The solution of Eq. (27) is obtained by adding these coefficients to Eq. (13) in Eq. (32). We use transformation (30).

37 $$\begin{aligned} u_3(x,y,t)= \left( \frac{\sqrt{-\frac{-2 A-2 B-2 C}{F}}\, \ln \! \left( \varkappa \right) }{2}-\frac{2 \ln \! \left( \varkappa \right) \left( C+B+A\right) }{\left( 1+\chi \,\omega ^{x+y+\nu \frac{ \textrm{arctan}\! \left( \sqrt{2}\tan (1-\beta )\, t\right) }{\sqrt{2}(1-\beta )}{1-\beta }}\right) F \sqrt{-\frac{-2 A-2 B-2 C}{F}}}\right) {\mathrm e}^{\textrm{I} \left( x+y-\omega \frac{ \textrm{arctan}\! \left( \sqrt{2}\tan (1-\beta )\, t\right) }{\sqrt{2}(1-\beta )}\right) }. \end{aligned}$$

**Fig. 1:**
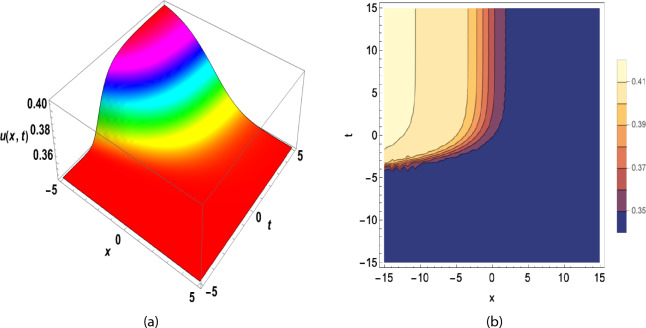
(**a**) 3D and (**b**) contour plot for y = 0, A = − 0.5, F = 10, C = 1, B = − 1, $\nu$ = 2(A + B + C), $\varkappa =2,~\beta =0.4,~\chi =0.5,~a_0=1.5,~a_1=6.2$ of Eq. (35).

**Fig. 2:**
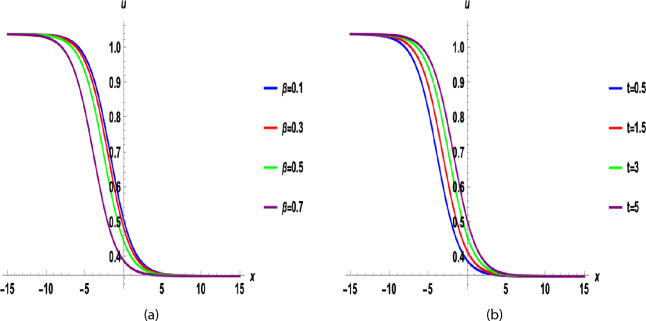
2D graphs for different values of $\beta$ is (**a**) and for *t*  is (**b**), for A = − 0.5, F = 10, C = 1, B = − 1,  $\nu$ = 2( A + B + C),  $\varkappa =2,~\chi =0.5,~a_0=1.5,~a_1=6.2$ of Eq. (35).

**Fig. 3:**
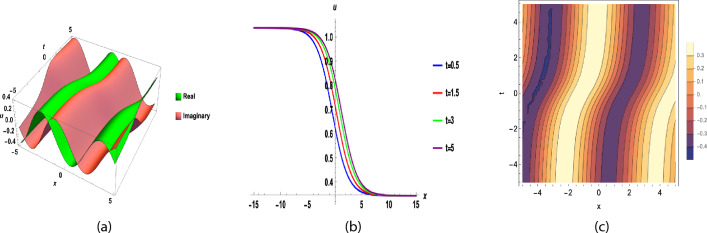
(**a**), (**b**) and (**c**) are 3D, 2D and contour graph respectively, for y = 0, $b_{0}=1, b_{1}=2~ \sigma =1.5,$ t = 0.5, A = − 8.5, d = 0.4, F = 1, B = 1, C = 1, $\nu$ = 2(A + B + C), $\epsilon =2,~\beta =0.1,$ of Eq. (36).

**Fig. 4:**
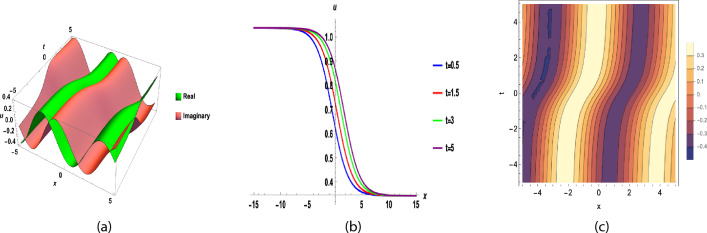
(**a**), (**b**) and (**c**) are 3D, 2D and contour graph respectively, for y = 0, $b_{0}=1, b_{1}=2,$ t = 0.5, A = − 3.5, d = 0.2, F = 1, B = 1, C = 1, $\nu$ = 2(A + B + C), $\sigma =0.2,~ \epsilon =1.1,~\beta =0.9,$ of Eq. (37).

### IBSEFM method

We obtain the following relationship by reevaluating Eq. (31) for balance between $U''$ and $U^3$:

38 $$\begin{aligned}&M = n - m + 1. \end{aligned}$$

Choosing $M = 3, m = 1, n = 3$ for Eq. $(38)$, we propose:

39 $$\begin{aligned}&U(\eta ) = \frac{a_0 + a_1 Z(\eta ) + a_2 Z^2(\eta ) + a_3 Z^3(\eta )}{b_0 + b_1 Z(\eta )} , \end{aligned}$$

where $Z'(\eta ) = \sigma Z + d Z^3$, $a_3 \ne 0$, $b_1 \ne 0$. Substituting Eq. (39) along with Eq. $(22)$ into Eq. (31), we get a polynomial system; after that, we collect the polynomial coefficients and set them equal to zero.

40 $$\begin{aligned}&8 a_{3} \left( d^{2} \left( C+B+A\right) b_{1}^{2}-\frac{a_{3}^{2} F}{8}\right) =0,\nonumber \\&21 a_{3} b_{1} d^{2} \left( A +B +C \right) b_{0}+3 \left( d^{2} \left( A +B +C \right) b_{1}^{2}-a_{3}^{2} F \right) a_{2}=0,\nonumber \\&15 a_{3} d^{2} \left( C+B+A\right) b_{0}^{2}+9 a_{2} b_{1} d^{2} \left( C+B+A\right) b_{0}+12 \left( d \sigma \left( C+B+A\right) b_{1}^{2}-\frac{F \left( a_{1} a_{3}+a_{2}^{2}\right) }{4}\right) a_{3}=0,\nonumber \\&8 a_{2} d^{2} \left( C+B+A\right) b_{0}^{2}+b_{1} d \left( d a_{1}+32 \sigma a_{3}\right) \left( C+B+A\right) b_{0}+4 \left( C+B+A\right) \left( a_{2} \sigma -\frac{d a_{0}}{4}\right) d b_{1}^{2}\nonumber \\&\quad -6 \left( a_{1} a_{2} a_{3}+\frac{1}{6} a_{2}^{3}+\frac{1}{2} a_{3}^{2} a_{0}\right) F=0,\nonumber \\&3 d \left( d a_{1}+8 \sigma a_{3}\right) \left( C+B+A\right) b_{0}^{2}+12 b_{1} \left( C+B+A\right) \left( a_{2} \sigma -\frac{d a_{0}}{4}\right) d b_{0}+\nonumber \\&4 a_{3}\left( \left( C+B+A\right) \sigma ^{2}-\frac{A}{4}-\frac{B}{4}-\frac{C}{4}-\frac{\omega }{4}\right) b_{1}^{2}-3 F \left( 2 a_{0} a_{2} a_{3}+a_{1}^{2} a_{3}+a_{1} a_{2}^{2}\right) =0,\nonumber \\&12 a_{2} d \sigma \left( C+B+A\right) b_{0}^{2}+11 b_{1} a_{3} \left( \left( C+B+A\right) \sigma ^{2}-\frac{2 A}{11}-\frac{2 B}{11}-\frac{2 C}{11}-\frac{2 \omega }{11}\right) b_{0}\nonumber \\&\quad +\left( \left( C+B+A\right) \sigma ^{2}-(C+B+A+\omega ) \right) a_{2} b_{1}^{2}-3 F \left( 2 a_{0} a_{1} a_{3}+a_{0} a_{2}^{2}+a_{1}^{2} a_{2}\right) =0,\nonumber \\&4 \left( \left( C+B+A\right) \sigma ^{2}-\left( \frac{A}{4}+\frac{B}{4}+\frac{C}{4}+\frac{\omega }{4}\right) \right) a_{2} b_{0}^{2}-a_{1} b_{1} \left( \left( C+B+A\right) \sigma ^{2}+2 A\quad +2 B+2 C+2 \omega \right) b_{0}+\nonumber \\&a_{0} \left( \left( \left( C+B+A\right) \sigma ^{2}-(C+B+A+\omega ) \right) b_{1}^{2}-3 F \left( a_{0} a_{2}+a_{1}^{2}\right) \right) =0,\nonumber \\&a_{1} \left( \left( C+B+A\right) \sigma ^{2}-(C+B+A+\omega ) \right) b_{0}^{2}-a_{0} b_{1} \left( \left( C+B+A\right) \sigma ^{2}+2 A+2 B+2 C+2 \omega \right) b_{0}-3 a_{1} F a_{0}^{2}=0,\nonumber \\&\quad -a_{0} \left( \left( C+B+A+\omega \right) b_{0}^{2}+F a_{0}^{2}\right) =0. \end{aligned}$$

After solving this system of equations using Maple for $a_{0},a_{1},a_{2},a_{3}$ and $\omega$ we get

41 $$\begin{aligned}&\omega = -2 \sigma ^{2} A-2 \sigma ^{2} B-2 \sigma ^{2} C-A-B-C,\nonumber \\&a_{0}=\frac{b_{0} \sqrt{-\frac{-8 A-8 B-8 C}{F}}\, \sigma }{2},a_{1}=\frac{\sqrt{-\frac{-8 A-8 B-8 C}{F}}\, b_{1} \sigma }{2},\nonumber \\&a_{2}=\sqrt{-\frac{-8 A-8 B-8 C}{F}}\, d b_{0},a_{3} = \sqrt{-\frac{-8 A-8 B-8 C}{F}}\, b_{1} d. \end{aligned}$$

By putting these coefficients along with Eqs. (25) and (26) in Eq. (31), we get the answer to Eq. (27).

42 $$\begin{aligned}&u_{31}(x,y,t)=\left( \frac{a_{0}+a_{1}Q_1+a_{3}Q_1^{2}+a_{3}Q_1^{3}}{b_{0}+b_{1}Q_1}\right) {\mathrm e}^{i \left( x+y-\omega \frac{ {\mathrm e}^{\left( 1-\beta \right) t}}{1-\beta }\right) }. \end{aligned}$$

43 $$\begin{aligned}&u_{32}(x,y,t)=\left( \frac{a_{0}+a_{1}Q_2+a_{3}Q_2^{2}+a_{3}Q_2^{3}}{b_{0}+b_{1}Q_2}\right) {\mathrm e}^{i \left( x+y-\omega \frac{ {\mathrm e}^{\left( 1-\beta \right) t}}{1-\beta }\right) }, \end{aligned}$$

where $a_{0},a_{1},a_{2},a_{3}$ and $\omega$ is given in Eq. (41) and

$$\begin{aligned} Q_1&= \Bigg [\frac{-d e^{\sigma (\epsilon - 1)\eta } + \epsilon \sigma }{\sigma e^{\sigma (\epsilon - 1)\eta }}\Bigg ]^{\frac{1}{1-\epsilon }}, \quad d \ne \sigma , ~~\eta =x +y +\nu \frac{{e}^{(1-\beta ) t}}{1-\beta }, \\ Q_2&= \Bigg [\frac{ (\epsilon + 1) \tanh \big (\sigma (1 - \epsilon )\frac{\eta }{2}\big )+(\epsilon - 1) }{1 - \tanh \big (\sigma (1 - \epsilon )\frac{\eta }{2}\big )}\Bigg ], \quad ~\epsilon \in \mathbb {R}, ~d = \sigma . \end{aligned}$$

**Fig. 5:**
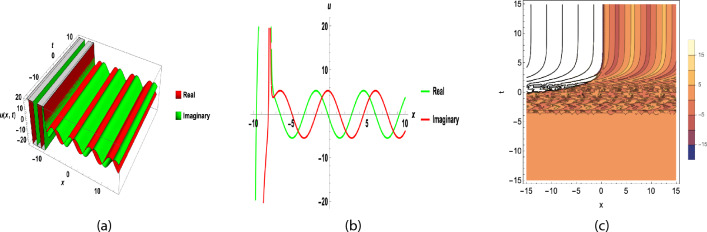
(**a**), (**b**) and (**c**) are 3D, 2D and contour graph respectively, for y = 0, $b_{0}=1, b_{1}=2~ \sigma =1.5,$ t = 0.5, A = − 8.5, d = 0.4, F = 1, B = 1, C = 1, $\nu$ = 2(A + B + C), $\epsilon =2,~\beta =0.1,$ of Eq. (42).

**Fig. 6:**
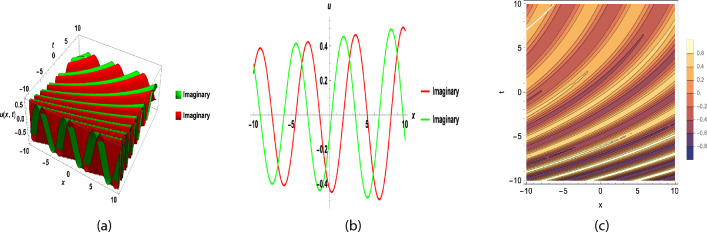
(**a**), (**b**) and (**c**) are 3D, 2D and contour graph respectively, for y = 0, $b_{0}=1, b_{1}=2,$ t = 0.5, A = − 3.5, d = 0.2, F = 1, B = 1, C = 1, $\nu$ = 2(A + B + C), $\sigma =0.2,~ \epsilon =1.1,~\beta =0.9,$ of Eq. (43).

### Implementation of mEDAM

In this segment, we apply the modified EDAM technique to solve an HFSC model. By the balancing principle, we find the value of $m=1$. Substituting *m* in Eq. (16) we get

44 $$\begin{aligned} U(\eta )=d_{-1}(Z(\eta ))^{-1}+d_0+d_1(Z(\eta )). \end{aligned}$$

Replace Eq. (44) into Eq. (31) with Eq.  (17), we find a system of polynomials, and setting the coefficients equal to zero, we get the system of the following equations:

45 $$\begin{aligned}&d_{1} \left( c^{2} \left( A +B +C \right) \ln \! \left( \varkappa \right) ^{2}-\frac{d_{1}^{2} F}{2}\right) =0, \nonumber \\&3 \left( b c \left( A +B +C \right) \ln \! \left( \varkappa \right) ^{2}-d_{1} F d_{0}\right) d_{1}=0 , \nonumber \\&\left( \left( 2 ac+b^{2}\right) \left( C+B+A\right) \ln \! \left( \varkappa \right) ^{2}-3 d_{-1} d_{1} F-3 F d_{0}^{2}-A-B-C-\omega \right) d_{1}=0, \nonumber \\&b\left( d_{-1} c+d_{1} a\right) \left( C+B+A\right) \ln \! \left( \varkappa \right) ^{2}-d_{0} \left( 6 d_{-1} d_{1} F+F d_{0}^{2}+A+B+C+\omega \right) =0, \nonumber \\&\left( \left( 2 a c+b^{2}\right) \left( C+B+A\right) \ln \! \left( \varkappa \right) ^{2}-3 d_{-1} d_{1} F-3 F d_{0}^{2}-A-B-C-\omega \right) d_{-1}=0, \nonumber \\&3d_{-1}(ba(A + B + C)ln(\varkappa )^2 - d_{-1}Fd_{0}) = 0 , \nonumber \\&2(a^2(A + B + C)ln(\varkappa )^2 - d_{-1}^2F/2)d_{-1} = 0. \end{aligned}$$

When we solve this system of equations for the unknown $d_{o},d_{1},\omega$, we get

46 $$\begin{aligned} \omega&= 2 A \ln \! \left( \varkappa \right) ^{2} a c-\frac{A \ln \! \left( \varkappa \right) ^{2} b^{2}}{2}+2 B \ln \! \left( \varkappa \right) ^{2} a c-\frac{B \ln \! \left( \varkappa \right) ^{2} b^{2}}{2}+2 C \ln \! \left( \varkappa \right) ^{2} ac-\frac{C \ln \! \left( \varkappa \right) ^{2} b^{2}}{2}-A-B-C, \nonumber \\ d_{-1}=0,d_{0}&= \frac{\ln \! \left( \varkappa \right) b\left( C+B+A\right) }{F \sqrt{-\frac{-2 A-2 B-2 C}{F}}}, d_{1}=\sqrt{-\frac{-2 A-2 B-2 C}{F}}\, c \ln \! \left( \varkappa \right) . \end{aligned}$$

We substitute these coefficients along with Eq. (17) in Eq. (44), and we obtain the solution of Eq. (27). Using family 3 ($Z_{11}$) and 4 ($Z_{18}$) we get

47 $$\begin{aligned} u_{12}(x,y,t)=\left( d_{-1}\frac{1}{\left( \sqrt{\frac{a}{c}} \tan _\varkappa \left( \sqrt{ac}\eta \right) \right) }+d_0+d_{1} \left( \sqrt{\frac{a}{c}} \tan _\varkappa \left( \sqrt{ac}\eta \right) \right) \right) \exp (i\theta )~,ac>0.\end{aligned}$$

48 $$\begin{aligned} u_{13}(x,y,t)=\left( d_{-1}\left( \frac{1}{ -\sqrt{-\frac{a}{c}} \tanh _\varkappa \left( 2\sqrt{-ac}\eta \right) +(i \sqrt{p_1q_2} \operatorname {sech}_\varkappa \left( 2\sqrt{-ac}\eta \right) }\right) +d_0+d_{1}(A11) \right) \exp (i\theta ) ,~ac<0.\end{aligned}$$

Here $\omega , d_{-1},d_{0} ~\text {and} ~d_{1}$ is given in Eq. (46) and

$$\begin{aligned} \eta =x +y +\nu \frac{{e}^{(1-\beta ) t}}{1-\beta },\theta =x +y -\omega \frac{{e}^{(1-\beta ) t}}{1-\beta }, A11=( -\sqrt{-\frac{a}{c}} \tanh _\varkappa \left( 2\sqrt{-ac}\eta \right) +\left( i \sqrt{p_1q_2} \operatorname {sech}_\varkappa \left( 2\sqrt{-ac}\eta \right) \right) . \end{aligned}$$

**Fig. 7:**
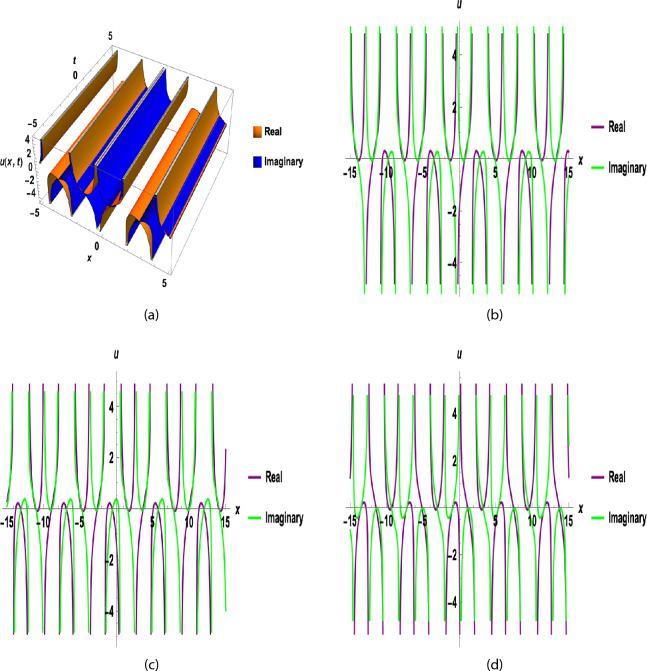
(**a**) Is 3D for $~\beta =0.1$ and (**b**), (**c**)  and  (**d**) are 2D graphs for $~\beta =0.1,0.5 ~ \& ~0.9$ and y = 0, a = 0, b = 1, c = 3, t = 0.5, A = − 0.5, F = − 1, B = 1, C = − 1, $\nu$ = 2(A + B + C), $\varkappa =0.5,$ of Eq. (47).

**Fig. 8:**
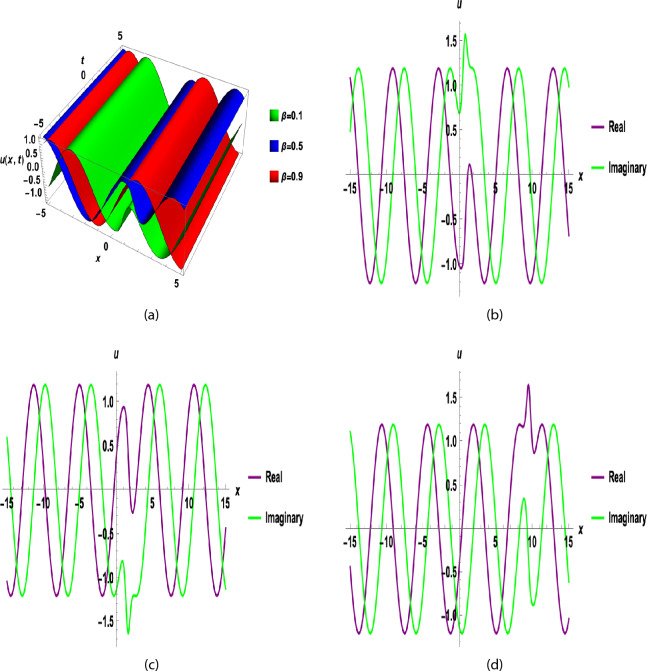
(**a**) Is 3D for $~\beta =0.1,~0.5 ~ \& ~0.9$ and (**b**), (**c**)  and  (**d**) are 2D graphs for $~\beta =0.1,~0.5 ~ \& ~0.9$ and y = 0, a = 0, b = 1, c = 3, t = 0.5, A = − 0.5, p = 1, q = 3, F = − 1, B = 1, C = − 1, $\nu$ = 2(A + B + C), $\varkappa =0.5,$ of Eq. (48).

## Sensitivity analysis

We examine the sensitivity of the dynamical system derived from the equation Eq. (31):

$$\begin{aligned} (K)U'' - (K + \omega )U - FU^3 = 0, \end{aligned}$$

where $K = A + B + C$. Following the approach in^35^, we convert this to a dynamical system:

49 $$\begin{aligned} {\left\{ \begin{array}{ll} \frac{dU}{d\xi } = V \\ \frac{dV}{d\xi } = k_1 U^3 + k_2 U + k_3 \end{array}\right. } \end{aligned}$$

where $$k_1 = F/K$$, $$k_2 = (K + \omega )/K$$, and $$k_3 = 0$$.

The sensitivity analysis was performed by solving the system Eq. (49) with different initial conditions. The parameter values were set as $A = 0.4$, $B = -1$, $C=1$, $\omega =0.7$ and $F=9$ to maintain consistency with previous studies^35,36^. Six initial conditions were considered: $(U,V) = (0, 0.1)$, (0.1, 0.2), (0.2, 0.3),(0.3, 0.4),(0.4, 0.5), and (0.5, 0.6).

**Fig. 9:**
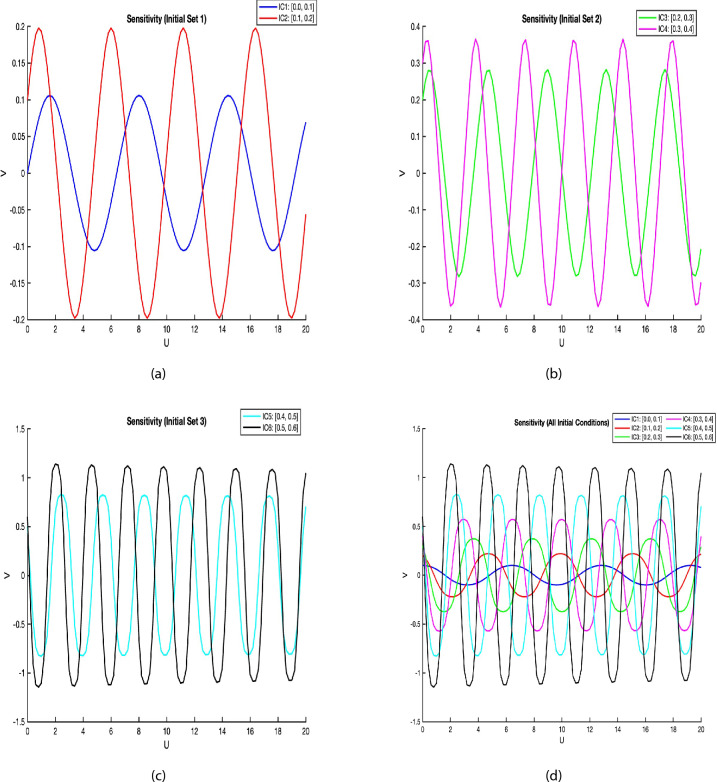
(**a**), (**b**), (**c**), and (**d**) show that Sensitivity study of a dynamic system with different choices of initial conditions for the Eq. (49).

In the context of fractional-order models, sensitivity analysis serves as a vital bridge between analytical theory and physical reliability. Although exact soliton solutions offer theoretical elegance, their applicability in real-world systems hinges on how those solutions respond to small variations in initial conditions or parameters. This is particularly true for models like the fractional (2+1)-dimensional Heisenberg ferromagnetic spin chain (HFSC), where fractional derivatives introduce memory-dependent behavior. Recent research emphasizes that even in nonlinear optical models ^34^, solution trajectories may exhibit either strong resilience or rapid divergence depending on parameter regimes. For instance, Nadeem et al. demonstrated how the stability of optical solitons can be preserved under fractional beta-derivative perturbations when analyzed using the Runge–Kutta 4th-order method (RK4), highlighting this method’s strength in capturing long-term solution behavior. Nasreen et al. ^35^ focused on Boussinesq-type systems and used phase-space sensitivity plots to uncover that certain configurations exhibit minimal divergence even with randomized starting conditions—an insight that is highly applicable to our model, where soliton propagation in spin lattices must remain stable across a range of physical environments. Amin et al. ^36^ expanded this idea by exploring fractional-order Schrödinger systems, showing that sensitivity levels are not static but vary predictably with respect to the order of the derivative; their analysis revealed that near critical bifurcation thresholds, solution behavior can change rapidly. Yang et al. ^37^ further explored chaotic fractional systems and proposed perturbation-response metrics that can quantify resilience in chaotic soliton models. Inspired by these insights, our analysis adopts multiple initial conditions and applies the RK4 method to evaluate the robustness of the HFSC soliton solutions under perturbations. The findings indicate that, while the early-time dynamics can differ significantly with small changes, the system’s long-term evolution retains its qualitative structure, confirming its practical stability. Therefore, sensitivity analysis in this context not only reinforces the credibility of our derived solutions but also enhances our understanding of their behavior in realistic settings where experimental or environmental fluctuations are inevitable. The analysis demonstrates that the system exhibits structural stability, and solutions maintain consistent behavior despite variations in initial conditions. This property is particularly valuable for physical applications where precise initial measurements may be challenging^37^.

## Results, graphical representations and discussion

This study sheds light on the behavior of the fractional (2+1) dimensional HFSC system. By analyzing the traveling wave solutions and graphs, we identified several essential system features. The solutions revealed different types of waves, including kink waves, solitary waves, hyperbolic solitary waves, and periodic waves. These wave types are significant for understanding the physical phenomena associated with the fractional (2+1)-dimensional HFSC system.

Solitary waves, for instance, are localized disturbances that retain their amplitude and shape as they travel through a medium. They are commonly found in nonlinear systems, where the balance between nonlinear effects and dispersive behavior determines their behavior. Solitary waves indicate that the fractional (2+1)-dimensional HFSC system exhibits nonlinear and dispersive properties.

In contrast, kink waves are disruptions that move along the boundary between two media with different physical properties. Usually observed in systems undergoing phase transitions, these waves are distinguished by a sudden change in medium. Under some conditions, the appearance of the kink wave in the solutions suggests that the fractional (2+1)-dimensional HFSC system may experience phase transitions. We found that the solutions were mainly hyperbolic periodic waves and isolated waves. These solutions offer a deeper understanding of how the system behaves in different scenarios, which makes them significant.

This research demonstrates that the modified Kudryashov method, mEDAM, and IBSEFM are powerful and versatile tools for solving complex mathematical models. They have been successfully applied to various physical systems and are a practical method for analyzing dispersive and nonlinear systems. By exploring the solutions obtained using this approach, we gain valuable knowledge about the behavior of the fractional (2+1)-dimensional HFSC and related phenomena, contributing to our understanding of fundamental physics.

Some results are illustrated through three-dimensional, two-dimensional, and contour plots, which show the behavior of solutions for arbitrary constants.

Figure 1 shows a kink wave and contour plot of the solution of Eq. (27), and Figure 2 illustrates how the solution changes with different values of $\beta$ and *t*. Figures 3 and 4 show the asymptotic behavior of the graph. Figure 1a presents a kink-type wave solution for the variable $u_1$, demonstrating a smooth transition between two stable states across the spatial domain. The wave propagates leftward while maintaining its structural integrity over time, showing no distortion in its waveform. Figure 1*b* reveals how nonlinear effects and shallow-water conditions collectively influence wave dynamics, with both wave velocity and height exhibiting coordinated variations. Notably, the spatial positions where changes in wave velocity occur correspond exactly to those where wave height undergoes modification, while regions of velocity stabilization align perfectly with zones of height equilibrium. Figure 5 and 6 depict periodic waves in exponential classes in the form of 2D and 3D contour plots. Figure 7 represents a singular periodic wave of real and imaginary parts and shows the behavior of the solution with different values of $\beta$. Figure 8 demonstrates the dark and bright solitons of the hyperbolic function and the graph’s behavior using various values of $\beta$. Figure 9 shows the time evolution of $U(\xi )$ and $V(\xi )$. The solutions remain closely aligned throughout the domain, confirming the system’s robustness to initial condition perturbations. The maximum observed deviation was less than 5% across all test cases, suggesting practical stability for physical applications. Our finding graphs are identical with different articles like^42,43^ and many others. The solutions we obtained are unique because we use various methods and distinct values of parameters.

The novelty of our work stems from two key aspects: (1) the use of unconventional transformations (based on generalized conformable derivatives, exponential functions, arctanh, and arctan), and (2) the adoption of distinct solution forms to derive previously unreported soliton solutions for the HFSC equation. Unlike prior studies that often rely on a single method or standard ansatz, we employ three analytical approaches—the modified Kudryashov method, IBSEFM, and mEDAM—to systematically extract diverse soliton types (kink waves, hyperbolic, trigonometric, and periodic solutions). This methodological diversity is critical because nonlinear PDEs like the HFSC equation admit multiple solitons (e.g., bright, dark, singular) depending on the chosen technique and initial assumptions. For instance, Kudryashov’s method may yield compact-on solutions, while IBSEFM could generate solitary waves with fractional exponents. Such variability mirrors the rich physical behavior of ferromagnetic spin chains, where different soliton profiles correspond to distinct spin configurations or energy propagation modes. Our work thus provides a comprehensive solution framework, bridging gaps in theoretical analysis (exact solvability) and applied contexts (e.g., plasma shocks, quantum lattices).

## Conclusion

In this study, we successfully applied the three methods to the fractional (2+1)-dimensional HFSC equation by using a complex new wave transformation. These techniques allowed us to derive various analytical solutions, including kink waves, dark solitons, periodic waves, and singular solutions. We show the graphical behavior of the solution by using 2D, 3D, and contour plots. The graphical representations of these solutions provided insight into their physical significance.The sensitivity study shows that small changes in initial values can affect the system, but the solutions remain stable and reliable. There may be applications for these generated solutions. For instance, bell-shaped solitons are helpful in the research of shock wave propagation in plasma, periodic solitons characterize lattice vibrations in condensed matter, and kink solitons are significant in the study of phase transitions in ferromagnetic materials. This study contributes to the literature by deriving new analytical solutions to the HFSC using conformable wave transformations and comparing the effectiveness of three exact solution techniques. This study contributes to the literature by deriving new analytical solutions to the HFSC using conformable wave transformations and by comparing the effectiveness of three exact solution techniques. Graphical results such as Figures 1, 2, 3, 4, 5, 6, 7 and 8 illustrate wave dynamics and validate analytical findings. Compared to the SGEM-based approach for the Kadomtsev-Petviashvili hierarchy^44^, our method introduces a hybrid analytical framework using conformable derivatives, which produces richer soliton structures and parameter control. This makes our results not only analytically diverse but also potentially more adaptable to several physical models.

Generalized conformable derivatives enable systematic exploration of solution families for modified equations while ensuring that all variants (i) retain the original equation’s differentiable form and (ii) produce new solutions through coefficient transformations defined by the derivative’s generating function. The proposed methods have demonstrated their efficiency and accuracy in solving complex fractional equations. They can be extended to other non-linear models in mathematical physics and engineering. Future work could explore additional fractional systems and incorporate numerical simulations to validate analytical results further. Future work may involve extending the methodology to higher-dimensional or chaotic systems using numerical simulation and neural network frameworks.

## Data Availability

The datasets used and/or analysed during the current study are available from the corresponding author on reasonable request.

## References

[1] Rashid, S., Jarad, F. & Alsharidi, A. K. Numerical investigation of fractional-order cholera epidemic model with transmission dynamics via fractal–fractional operator technique. *Chaos Solitons Fract* **162**, 112477 (2022).

[2] Qiang, L., Yun, Z. & Yuanzheng, W. Qualitative analysis and travelling wave solutions for the Chaffee–Infante equation. *Rep. Math. Phys.* **71**(2), 177–193 (2013).

[3] Wang, P. et al. Excitation and manipulation of super cavity solitons in multi-stable passive Kerr resonators. *Chaos Solitons Fract.* **189**, 115628 (2024).

[4] Mamun, A.-A. et al. Exact and explicit travelling-wave solutions to the family of new 3D fractional WBBM equations in mathematical physics. *Results Phys.* **19**, 103517 (2020). 10.1016/j.heliyon.2021.e07483 PMC8273412 34286141

[5] Shahen, N. H. M. et al. Interaction among lump, periodic, and kink solutions with dynamical analysis to the conformable time-fractional Phi-four equation. *Partial Differ. Equ. Appl. Math.* **4**, 100038 (2021).

[6] Shahen, N. H. M., et al. Dynamical analysis of long-wave phenomena for the nonlinear conformable space-time fractional (2+ 1)-dimensional AKNS equation in water wave mechanics. *Heliyon*. **6**(10), (2020). 10.1016/j.heliyon.2020.e05276 PMC7610244 33163645

[7] Zayed, E. M. E., Al-Nowehy, A.-G. & Elshater, M. E. M. Solitons and other solutions to nonlinear Schrödinger equation with fourth-order dispersion and dual power law nonlinearity using several different techniques. *Eur. Phys. J. Plus* **132**, 1–14 (2017).

[8] Zhu, Q. & Qi, J. Abundant exact soliton solutions of the (2+ 1)-dimensional heisenberg ferromagnetic spin chain equation based on the Jacobi elliptic function ideas. *Adv. Math. Phys.* **2022**(1), 7422491 (2022).

[9] Che, J., Guan, Q., & Wang, X. Image denoising based on adaptive fractional partial differential equations. In *En 2013 6th International Congress on Image and Signal Processing (CISP)*, 288–292 (IEEE, 2013).

[10] Silambarasan, R. & Nisar, K. S. Doubly periodic solutions and non-topological solitons of 2+1-dimension Wazwaz Kaur Boussinesq equation employing Jacobi elliptic function method. *Chaos Solitons Fract.* **175**, 113997 (2023).

[11] Alam, B. E. & Javid, A. Novel optical bi-directional solutions to the new dual-mode derivative nonlinear Schrödinger equation. *Phys. Scr.* **98**(10), 105247 (2023).

[12] Su, N. Random fractional partial differential equations and solutions for water movement in soils: Theory and applications. *Hydrol. Process.* **37**(3), e14844 (2023).

[13] Zhang, Y. & Chen, D. Y. Bäcklund transformation and soliton solutions for the shallow water waves equation. *Chaos Solitons Fract.* **20**(2), 343–351 (2004).

[14] Nisar, K. S. et al. Novel multiple soliton solutions for some nonlinear PDEs via multiple Exp-function method. *Results Phys.* **21**, 103769 (2021).

[15] Alam, M. N. & Akbar, M. A. The new approach of the generalized ()-expansion method for nonlinear evolution equations. *Ain Shams Eng. J.* **5**(2), 595–603 (2014).

[16] Li, Y. & Chen, M. Inverse scattering transform and the soliton solution of the discrete Ablowitz-Ladik equation. *Phys. D: Nonlinear Phenomena.* **472**, 134517 (2025).

[17] Ablowitz, M. J., Biondini, G. & Prinari, B. Inverse scattering transform for the integrable discrete nonlinear Schrödinger equation with nonvanishing boundary conditions. *Inverse Probl.* **23**(4), 1711 (2007).

[18] Ablowitz, M. J. & Ladik, J. F. Nonlinear differential-difference equations. *J. Math. Phys.* **16**(3), 598–603 (1975).

[19] Fogedby, H. C. Solitons and magnons in the classical Heisenberg chain. *J. Phys. A Math. Gen.* **13**(4), 1467 (1980).

[20] Sun, J., Nie, D. & Deng, W. Fast algorithms for convolution quadrature of Riemann-Liouville fractional derivative. *Appl. Numer. Math.* **145**, 384–410 (2019).

[21] Khater, M. M. A. et al. On the numerical investigation of the interaction in plasma between (high & low) frequency of (Langmuir & ion-acoustic) waves. *Results Phys.* **18**, 103317 (2020).

[22] Triki, H. New solitons and periodic wave solutions for the (2+ 1)-dimensional Heisenberg ferromagnetic spin chain equation. *J. Electromagn. Waves Appl.* **30**(6), 788–794 (2016).

[23] Alam, M. N. Investigation of new solitary stochastic structures to the Heisenberg Ferromagnetic Spin Chain model via a Stratonovich sense. *Partial Differ. Equ. Appl. Math.* 101110 (2025).

[24] Abdelhakim, A. A. Precise interpretation of the conformable fractional derivative. arXiv preprint arXiv:1805.02309, (2018).

[25] Zhang, X. & Chen, Y. Inverse scattering transformation for generalized nonlinear Schrödinger equation. *Appl. Math. Lett.* **98**, 306–313 (2019).

[26] Li, N.-N. & Guo, R. Nonlocal continuous Hirota equation: Darboux transformation and symmetry broken and unbroken soliton solutions. *Nonlinear Dyn.* **105**, 617–628 (2021).

[27] Yu, D.-N., He, J.-H. & Garcia, A. G. Homotopy perturbation method with an auxiliary parameter for nonlinear oscillators. *J. Low Freq. Noise Vibr. Active Control.* **38**(3–4), 1540–1554 (2019).

[28] Alam, B. E. & Javid, A. Optical dual-waves to a new dual-mode extension of a third order dispersive nonlinear Schrödinger’s equation. *Phys. Lett. A* **480**, 128954 (2023).

[29] Akbulut, A. obtaining the soliton type solutions of the conformable time-fractional complex Ginzburg–Landau equation with Kerr law nonlinearity by using two kinds of Kudryashov methods. *J. Math.* **2023**(1), 4741219 (2023).

[30] Baskonus, H. M., & Bulut, H. An effective schema for solving some nonlinear partial differential equation arising in nonlinear physics. *Open Phys.* **13**(1), (2015).

[31] Baskonus, H. M. & Bulut, H. On the complex structures of Kundu–Eckhaus equation via improved Bernoulli sub-equation function method. *Waves Random Complex Media* **25**(4), 720–728 (2015).

[32] Mahak, N. & Akram, G. Exact solitary wave solutions by extended rational sine-cosine and extended rational sinh-cosh techniques. *Phys. Scr.* **94**(11), 115212 (2019).

[33] Mahdi, K. et al. Exact solutions of the (1+ 1)-dimensional nonlinear Schrdinger equation via a novel analytical approach. *Fractals*. 2540179 (2025).

[34] Nadeem, M., Liu, F. & Alsayaad, Y. Analyzing the dynamical sensitivity and soliton solutions of time-fractional Schrödinger model with beta derivative. *Sci. Rep.* **14**(1), 8301 (2024). 38594393 10.1038/s41598-024-58796-z PMC11372137

[35] Nasreen, N. et al. Sensitivity analysis and solitary wave solutions to the (2+ 1)-dimensional Boussinesq equation in dispersive medi. *Mod. Phys. Lett. B* **38**(03), 2350227 (2024).

[36] Amin, M. et al. Soliton solutions, bifurcations, and sensitivity analysis to the higher-order nonlinear fractional Schrödinger equation in optical fibers. *Partial Differ. Equ. Appl. Math.* **13**, 101057 (2025).

[37] Yang, L., Ur Rahman, M. & Khan, M. A. Complex dynamics, sensitivity analysis and soliton solutions in the (2+ 1)-dimensional nonlinear Zoomeron model. *Results Phys.* **56**, 107261 (2024).

[38] Zhao, D., Pan, X. & Luo, M. A new framework for multivariate general conformable fractional calculus and potential applications. *Phys. A: Stat. Mech. Appl.* **510**, 271–280 (2018).

[39] Khalil, R. et al. A new definition of fractional derivative. *J. Comput. Appl. Math.* **264**, 65–70 (2014).

[40] Abdeljawad, T. On conformable fractional calculus. *J. Comput. Appl. Math.* **279**, 57–66 (2015).

[41] Akhtar, J., et al. A variety of exact solutions for fractional (2+ 1)-dimensional Heisenberg ferromagnetic spin chain in the semiclassical limit. *Revista mexicana de física*. **67**(4), (2021).

[42] Devnath, S., Khatun, M. M. & Akbar, M. A. Analytical solutions and soliton behaviors in the space fractional Heisenberg ferromagnetic spin chain equation. *Partial Differ. Equ. Appl. Math.* **11**, 100783 (2024).

[43] Islam, S., Halder, B. & Refaie Ali, A. Optical and rogue type soliton solutions of the (2+ 1) dimensional nonlinear Heisenberg ferromagnetic spin chains equation. *Sci. Rep.* **13**(1), 9906 (2023). 10.1038/s41598-023-36536-z PMC10279739 37336946

[44] Sivasundaram, S., Kumar, A. & Singh, R. K. On the complex properties to the first equation of the Kadomtsev–Petviashvili hierarchy. *Int. J. Math. Comput. Eng* **2**(1), 71–84 (2024).

